# Thomas Blizard

**Published:** 1838-07

**Authors:** 


### OBITUARY.

### THOMAS BLIZARD, ESQ.

On the 7th of May last, at his house in Cumberland terrace, Regent's Park, aged sixty-six. Mr. Blizard commenced his professional life by being apprenticed at Surgeon's Hall, in 1789, and he obtained the greater portion of his education at the London Hospital, to which his cousin, Sir W. Blizard, was attached. Having received his diploma, he was elected assistant-surgeon to that hospital in 1795, and he at once commenced the system of teaching anatomy in its school. In 1796 he was appointed professor of anatomy to the corporation of surgeons, and delivered lectures in that capacity for three years. In 1797 he became surgeon to the London Hospital. He now resigned his anatomical lectureship; for his time was already fully occupied in the duties of the hospital and his increasing private practice. To these he applied himself with the most unremitting zeal, daily learning in the one the means, and earning in the other the reward, of bestowing benefits on both. From this time his practice increased every year; and, in 1816, finding it occupy every hour that he could, consistently with health and comfort, bestow upon it, he resigned the surgeoncy to the London Hospital. In the same year he was chosen one of the council of the College of Surgeons; and in the following, when his practice in the preceding six months had produced him a larger sum than is recorded of any medical practitioner, he resigned every professional occupation, and retired to the continent. The cause of this sudden retirement was the bad health of Mrs. Blizard, to the care and solace of whom the whole of his future life was devoted, with a tenderness and vigilance almost unexampled. In 1837, death at last deprived him of his only care, and broke almost the last tie which had held him to the earth. But this tie, painful as it had been, seemed necessary to his life. From the time of Mrs. Blizard's decease, one might see an evident alteration in his manner and appearance: he was no longer anxious to live; and, when his last illness seized him, he rapidly, and as it were involuntarily, succumbed to it.

Mr. Blizard was equally distinguished as a scientific and practical surgeon, and as a member of society. One who was long a pupil at the London Hospital says, that the opinion which he formed of him from observing his practice was, that he saw further, saw clearer, and acted more energetically than any surgeon he ever knew. His surgery was indeed purely practical: his anatomical knowledge was so exact, that no anticipated difficulty of manipulation deterred him from an operation when he deemed it necessary; nor did any unexpected accident embarrass him in its performance. Nay, even after twenty years of retirement, his anatomy was sitll good and clear; as easily producible as ever in discussing a case, or examining a preparation; no rust had accumulated on its polished and well-tempered, though long-neglected surface. The excellence of his lithotomy, which he always performed with the long and slender-beaked knife which still bears his name, has always been enthusiastically described by his old pupils: yet he was not fond of operating, and so little anxious to display his own powers, that he was always as ready to assist others in their difficulties as to take the chief part himself. Mild, and even affectionate, as his manners were towards his patients, and in the extensive circle of his friends, his spirit was truly indomitable when he was opposed in the pursuit of what he deemed to be just and honorable. His success was as little the result of servility as of private interest; he fairly and honestly worked his own way, and often against the tide; repelled by no obstacle, disturbed by no fear, retarded by no discouragement or indolence; entirely by his own industry he gained the knowledge which first brought him into public notice; and, by the unremitted exertion with which he pursued every advantage when once attained, he had earned by his profession, and at the age of forty-six, an ample fortune.*

* Med. Gazette, June 9, 1838.

